# Associations between Food Insecurity and Supplemental Nutrition Assistance Program (SNAP) participation with ultra-processed food intake in lower-income U.S. adolescents

**DOI:** 10.1017/jns.2025.24

**Published:** 2025-06-04

**Authors:** Aarohee P. Fulay, Ana Baylin, Julia A. Wolfson, Joyce M. Lee, Euridice Martinez-Steele, Cindy W. Leung

**Affiliations:** 1 Department of Epidemiology, University of Pittsburgh School of Public Health, Pittsburgh, PA, USA; 2 Department of Nutritional Sciences, University of Michigan School of Public Health, Ann Arbor, MI, USA; 3 Department of Epidemiology, University of Michigan School of Public Health, Ann Arbor, MI, USA; 4 Departments of International Health and Health Policy and Management, Johns Hopkins Bloomberg School of Public Health, Baltimore, MD, USA; 5 Susan B. Meister Child Health Evaluation and Research Center, Division of Pediatric Endocrinology, University of Michigan Medical School, Ann Arbor, MI, USA; 6 Department of Nutrition, School of Public Health, University of Sao Paulo, São Paulo, Brazil; 7 Center for Epidemiological Studies in Health and Nutrition, University of São Paulo, São Paulo, Brazil; 8 Department of Nutrition, Harvard T.H. Chan School of Public Health, Boston, MA, USA

**Keywords:** Adolescents, Food security, Supplemental nutrition assistance program (SNAP), Ultra-processed foods (UPF)

## Abstract

Ultra-processed foods (UPFs) have negative health consequences. Food insecurity and Supplemental Nutrition Assistance Program (SNAP) are associated with higher UPF intake in U.S. adults, but this has not been examined in U.S. adolescents. This study assesses associations between food security status and SNAP participation with UPF intake in 3,067 adolescents aged 12–19 years with household incomes at or below 300% of the federal poverty line from the 2007–2016 National Health and Nutrition Examination Survey. UPF is defined using the Nova classification and measured as a percentage of daily total energy intake (TEI). High food security, marginal food security, or food insecurity status was determined through the U.S. Department of Agriculture’s eighteen-item Household Food Security Survey. SNAP participation was deemed affirmative if the household reported receiving SNAP benefits in the last year. Multivariable linear regressions that controlled for TEI and sociodemographic covariates and accounted for the complex survey design examined associations between food insecurity and SNAP participation with UPF intake. In the sample, the prevalence of marginal food security was 15.9%, the prevalence of food insecurity was 33.8%, and the prevalence of SNAP participation was 36.5%. After multivariate adjustment, there were no significant differences in UPF intake by food security status. Adolescents participating in SNAP consumed 2.7% higher UPF intake (95% CI: 0.1%, 5.2%, p = 0.04) compared to adolescents not participating in SNAP. Among lower-income U.S. adolescents, SNAP participation but not food security status was associated with higher UPF intake. Programs and policies promoting the intake of more healthful, minimally processed foods should be strengthened.

## Introduction

Ultra-processed foods (UPFs) are defined as ‘formulations of ingredients, mostly of exclusive industrial use, that result from a series of industrial processes’.^(1)^ UPFs tend to be high in sodium, sugar, and saturated fat^(1)^ as well as low in fibre.^(2)^ Additionally, they have been shown to increase calorie intake^(3)^ and are hyper-palatable.^(1)^ UPFs have several negative health implications. In adults, UPF intake has been associated with increased risk of CVD,^(4)^ type 2 diabetes,^(5)^ and cancer.^(6)^ In adolescents, UPF intake has been associated with higher adiposity.^(7)^

UPFs are ubiquitous in the national food supply. For U.S. adults, on average, 55.4% of total energy intake (TEI) comes from UPFs.^(8)^ For U.S. adolescents, average UPF consumption is even higher, at 67.7%.^(9)^ Recent evidence suggests that certain sociodemographic characteristics^(10)^ such as education^(11)^ and race^(9)^ are associated with UPF intake. Furthermore, a recent study showed that two structural factors related to nutrition equity — (1) food insecurity and (2) Supplemental Nutrition Assistance Program (SNAP) participation — were associated with higher UPF intake in lower-income U.S. adults.^(12)^ However, the associations between food insecurity and SNAP participation with UPF intake in U.S. adolescents have not been sufficiently examined.

Food insecurity, defined as ‘a household-level economic and social condition of limited or uncertain access to adequate food,’^(13)^ is associated with poor dietary quality^(14,15)^ and higher UPF intake in lower-income U.S. adults.^(12)^ Initial evidence on food insecurity and dietary quality in U.S. adolescents was scarce but indicated possible associations with lower fruit, calcium, and iron intake.^(16)^ A 2020 review indicates that, among adolescents, food insecurity might be associated with higher intakes of certain food groups, such as sugar-sweetened beverages (SSB) and fast food.^(17)^

The SNAP is the largest federal nutrition assistance programme that provides lower-income Americans with money to purchase food.^(18)^ However, even with SNAP benefits, healthy foods are expensive and SNAP participants may need to prioritise purchases of low-cost, shelf-stable foods of lower nutritional quality.^(19,20)^ Participation in SNAP has been associated with 1.7% higher UPF intake^(12)^ and poor dietary quality^(21)^ among U.S. adults. For U.S. adolescents, evidence indicates that participation in SNAP is associated with lower quality dietary intake compared to adolescents who do not participate in SNAP.^(22–24)^

Overall, prior studies have examined associations between food insecurity and SNAP participation with dietary quality. However, UPF intake is an important outcome because it may more accurately capture certain foods that are overconsumed compared to other measures of dietary quality. For example, due to the top-coding of the added sugars, sodium, refined grains, and fatty acids components in the Healthy Eating Index (HEI) 2020, which compresses high and extremely high values of a nutrient into the same value, it might not sufficiently distinguish between high and extremely high consumption of these nutrients.^(25)^ However, to the best of our knowledge, no research to date has examined the association between household food insecurity and SNAP participation with UPF intake in U.S. adolescents, adjusted for key confounders. U.S. adolescents consume high levels of UPFs,^(9)^ which have been associated with adiposity^(7)^ and adverse cardiovascular health factors^(26)^ in this age group. Research that examines the potential associations of food security and SNAP participation with UPF intake could highlight possible intervention points to improve dietary intake and prevent negative health outcomes in this subpopulation.

We therefore examine the association between household food insecurity and SNAP participation with UPF intake in a national sample of lower-income (300% federal poverty line or below) U.S. adolescents aged 12–19 years from the 2007–2016 National Health and Nutrition Examination Survey (NHANES). We also examine the interaction between food insecurity and SNAP participation. We hypothesised that household food insecurity and SNAP participation would both be associated with higher adolescent UPF intake. We also hypothesised that there would be a statistically significant interaction between food insecurity and SNAP.

## Methods

### Data source

The data were obtained from NHANES, a nationally representative, complex, continuous cross-sectional survey that releases data on demographic information, dietary intake, and health in 2-year cycles.^(27)^ In order to maintain comparability with a parallel paper, which examines UPF intake in lower-income U.S. adults,^(12)^ this paper uses data from 2007 to 2016.

### Participants

**Fig. 1.**
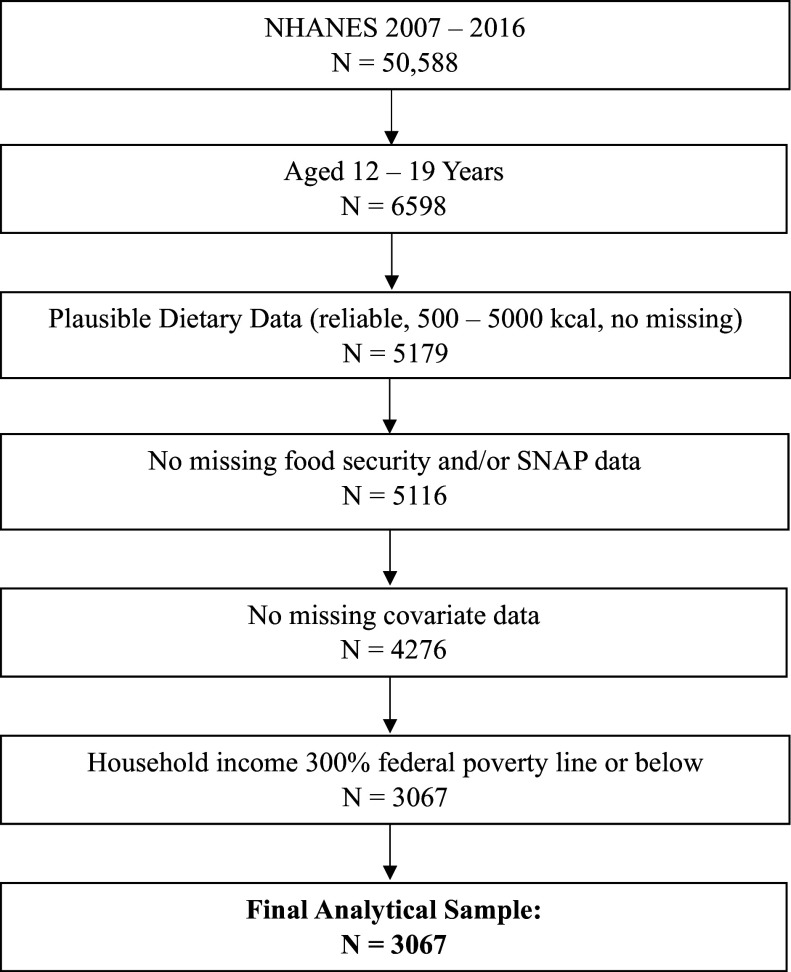
Flowchart for NHANES analytical sample (n = 3067).

Participants were included if they had information on the exposures, outcome, and covariates of interest. For this analysis, individuals were included in the sample if they completed two 24-h dietary recalls, which were ‘reliable and met the minimum criteria’ according to NHANES documentation.^(27)^ The analysis was also restricted to individuals who reported consuming 500–5000 kilocalories; previous studies using NHANES data have used this range to indicate plausible energy intakes.^(15)^ Additionally, to limit confounding by income, the sample was restricted to 300% federal poverty line or below, as done in previous comparable analyses^(22,28)^. The final sample included 3067 U.S. adolescents aged 12–19 years with lower incomes (300% federal poverty line or below). More details about the construction of the analytical sample can be found in the flowchart (Fig. 1).

### Exposures

The exposures were household food security and household SNAP participation. Food security status was determined by the United States Department of Agriculture (USDA) eighteen-item Household Food Security Survey Module. Following USDA guidelines, households were classified as experiencing high food security (0 affirmative responses), marginal food security (1–2 affirmative responses), low food security (3–7 affirmative responses), or very low food security (8–18 affirmative responses).^(29)^ Marginal food security is defined as ‘one or two reported indications — typically of anxiety over food sufficiency or shortage of food in the house, with little or no indication of changes in diets or food intake’; low food security is defined as ‘reports of reduced quality, variety, or desirability of diet, with little or no indication of reduced food intake’; and very low food security is defined as ‘reports of multiple indications of disrupted eating patterns and reduced food intake’.^(13)^ For this analysis, low food security and very low food security were grouped together, as the USDA considers food insecurity to include both these categories.^(13)^ Thus, we created a three-category variable: 1 = high food security, 2 = marginal food security, and 3 = food insecurity. Household SNAP participation was deemed affirmative if anyone in the household had reported receiving SNAP benefits in the past 12 months.^(27)^

### Outcome

The outcome was UPF intake as a percentage of daily TEI. The Nova classification system^(1)^ was used to classify foods as UPFs or non-UPFs. This system has been previously applied to NHANES data with detailed methods provided elsewhere.^(30,31)^ In brief, NHANES collects dietary data through 24-h dietary recalls that provide detailed information on the food items consumed.^(27)^ The first 24-h dietary recall is administered in-person at a mobile examination clinic where individuals 12 years of age and older self-report foods and beverages consumed within the last 24 h to a trained interviewer. The second recall is administered over the phone 3–10 d later.^(27)^

The NHANES dietary data were linked to the USDA’s Food and Nutrient Database for Dietary Studies (FNDDS) data^(32)^ by food codes. The FNDDS data contains food codes and standard reference (SR) codes, and a re-merging process has been applied to this dataset to disaggregate the food codes into their underlying SR codes. Based on the descriptions provided for FNDDS food codes and SR codes, food codes were classified as UPFs or non-UPFs. For all food codes judged to be a handmade recipe, the classification was applied to the underlying SR codes. Then, UPF intake as a percentage of TEI was calculated by dividing the number of calories from UPFs by the total number of calories consumed per day by an individual. The data were averaged across the 2 d.

### Covariates

Based on known associations with the exposures and the outcome, we determined covariates to include in our analyses to account for potential confounding. The covariates of interest were adolescent age (in years), sex (male/female), race/ethnicity, sedentary time (<=6 h or >6 h/d), vigorous recreational activity (yes/no), moderate recreational activity (yes/no), household income-to-poverty ratio, household respondent marital status (married/partnered or single/unpartnered), and household respondent educational attainment (high school graduate/not high school graduate). Race/ethnicity categories were non-Hispanic White, non-Hispanic Black, Mexican American, other Hispanic ethnicity, and other race. The household income-to-poverty ratio was determined by NHANES by dividing household income by the federal poverty guidelines.^(27)^ The household respondent is an adult member of the household who responded to some aspects of the survey on behalf of the household.^(27)^

### Statistical analysis

For descriptive statistics, weighted means and proportions were calculated. Simple linear regressions were used to assess differences in continuous variables, and Rao-Scott chi-square tests were used for categorical variables. For the main analyses, multivariable linear regressions were used to separately assess the associations between household food insecurity and SNAP participation with UPF intake. For both sets of analyses, Model 1 adjusted for age, sex, and TEI. For the food insecurity analyses, Model 2 adjusted for all covariates described above and TEI. The interaction of food insecurity and SNAP was tested by including the interaction term in the model. For the SNAP analyses, Model 2 adjusted for all covariates described above, TEI, and household food security status. Income was included as a linear and quadratic term to more accurately model the relationship between income and dietary quality. All analyses used survey procedures that accounted for the survey strata, clustering, and weights. All original 2-year dietary survey weights were recalculated to match the 10-year study period. The analyses were conducted in SAS Version 9.4 (SAS Institute, Cary, NC).

## Results

In this sample, 15.9% of lower-income U.S. adolescents experienced marginal food security, and 33.8% experienced food insecurity (Table 1). Adolescents with food insecurity were more likely to be part of a SNAP-participating household (p < 0.0001) and be non-Hispanic Black, Mexican American, or other Hispanic ethnicity (p < 0.0001). Adolescents with high food security were more likely to report engaging in vigorous recreational activity (p = 0.03). Adolescents with food insecurity were more likely to come from households that were lower-income (p < 0.0001), participating in SNAP (p < 0.0001), and where the household respondent was not married/partnered (p = 0.002) and not a high school graduate (p = 0.0002).

**Table 1.** Associations between household food insecurity and sociodemographic and health characteristics in a lower-income sample of adolescents aged 12–19 years in NHANES cycles 2007–2016^a^

	Full sample weighted % or mean	N or weighted SE	High food security weighted % or mean^b^	N or weighted SE	Marginal food security weighted % or mean^c^	N or weighted SE	Food insecurity weighted % or mean^d^	N or weighted SE	p-value
Overall (%)	100	3067	50.3	1409	15.9	527	33.8	1131	
Adolescent characteristics									
Age (years), mean (SE)	15.4	0.06	15.4	0.09	15.6	0.16	15.3	0.10	0.17
Female (%)	52.1	1557	52.6	711	57.2	293	49.1	553	0.17
Race/ethnicity (%)									**< 0.0001** ^*^
Non-Hispanic White	46.2	706	54.3	367	42.1	109	36.1	230	
Non-Hispanic Black	17.9	814	15.2	347	21.4	156	20.3	311	
Mexican American	20.2	853	15.7	355	20.5	136	26.7	362	
Other Hispanic ethnicity	8.6	391	7.5	171	10.3	76	9.4	144	
Other race/ethnicity	7.1	303	7.2	169	5.8	50	7.5	84	
Vigorous recreational activity in typical week (%Yes)	57.7	1716	60.7	823	50.4	275	56.6	618	**0.03** ^*^
Moderate recreational activity in typical week (%Yes)	52.4	1482	55.2	701	50.6	259	49.2	522	0.16
Sedentary time <= 6 h (%)	31.2	915	30.7	407	34.2	171	30.4	337	0.62
Household characteristics									
HH respondent education ≥ high school graduate (%)	70.3	1967	76.0	967	68.1	335	62.9	665	**0.0002** ^*^
HH respondent married/partnered (%)	62.8	1865	68.3	925	57.3	309	57.0	631	**0.002** ^*^
HH income-to-poverty ratio, mean (SE)^e^	1.42	0.03	1.67	0.04	1.32	0.07	1.09	0.04	**< 0.0001** ^*^
SNAP participation (%)	36.5	1238	23.8	393	40.3	240	53.5	605	**< 0.0001** ^*^

*Note:* NHANES, National Health and Nutrition Examination Survey; SE, standard error; HH, household; SNAP, Supplemental Nutrition Assistance Program.

a Analyses were conducted using survey procedures that take into account the complex survey design. Rao-Scott chi-squared tests were used for categorical variables, and linear regressions were used for continuous variables.

b United States Department of Agriculture (USDA) high food security definition: *no reported indications of food-access problems or limitations.* ^(13)^

c USDA marginal food security definition: *one or two reported indications — typically of anxiety over food sufficiency or shortage of food in the house. Little or no indication of changes in diets or food intake.* ^(13)^

d USDA food insecurity definition: *household-level economic and social condition of limited or uncertain access to adequate food*.^(13)^

e Income-to-poverty ratio: income divided by the federal poverty line.

* Statistically significant estimates at alpha = 0.05 are bolded.

Table 2 shows the associations between household food security status and adolescent UPF intake. For Model 1, which adjusted for age, sex, and TEI, the adjusted mean UPF intake was 66.3% of TEI for adolescents with high food security, 66.5% of TEI for adolescents with marginal food security (p-value = 0.92), and 65.7% of TEI for adolescents with food insecurity (p-value = 0.50). For Model 2, the adjusted mean intake was 64.4% of TEI for high food security, 64.5% of TEI for marginal food security (p-value = 0.92), and 64.1% of TEI for food insecurity (p-value = 0.83). Household food insecurity was not associated with adolescent UPF intake in either model.

**Table 2.** Linear regressions between food insecurity and ultra-processed food intake (as percentage of total energy) in lower-income (300% FPL and below) adolescents aged 12–19 years (n = 3067) in NHANES cycles 2007–2016^a^

	Model 1^b^					Model 2^c^				
	Adjusted mean	Estimate	95% CI LL	95% CI UL	p-value	Adjusted mean	Estimate	95% CI LL	95% CI UL	p-value
High food security	66.3	Ref.				64.4	Ref.			
Marginal food security	66.5	0.1	–2.7	3.0	0.92	64.5	0.2	–2.9	3.2	0.92
Food insecurity	65.7	–0.7	–2.7	1.3	0.50	64.1	–0.2	–2.4	1.9	0.83
p-for-trend					0.53					0.84

*Note:* FPL, federal poverty line; NHANES, National Health and Nutrition Examination Survey; CI, confidence interval; LL, lower limit; UL, upper limit.

a Analyses were conducted using survey procedures that take into account the complex survey design.

b Model 1 is adjusted for total energy intake, age, and sex.

c Model 2 is adjusted for total energy intake, adolescent age, sex, race/ethnicity, vigorous recreational activity, moderate recreational activity, sedentary time, household respondent education, marital status, and income (linear and quadratic term).

However, household SNAP participation was associated with higher UPF intake (Table 3). For Model 1, the adjusted mean UPF intake was 65.2% of TEI for SNAP non-participants and 67.7% of TEI for SNAP participants(p = 0.04). For Model 2, which adjusted for all covariates, TEI, and household food security status, the adjusted mean UPF intake was 63.2% of TEI for SNAP non-participants and 65.9% of TEI for SNAP participants; therefore, SNAP participation was associated with 2.7% higher UPF intake (p = 0.04). The interaction term between household food security status and household SNAP participation was not statistically significant (p = 0.48).

**Table 3.** Linear regressions between household SNAP participation and ultra-processed food intake (as percentage of total energy) in lower-income (300% FPL and below) adolescents aged 12–19 years (n = 3067) in NHANES cycles 2007–2016^a^

	Model 1^b^					Model 2^c^				
	Adjusted Mean	Estimate	95% CI LL	95% CI UL	p-value	Adjusted Mean	Estimate	95% CI LL	95% CI UL	p-value
SNAP non-participation	65.2	Ref.				63.2	Ref.			
SNAP participation	67.7	2.5	0.2	4.8	**0.04** ^*^	65.9	2.7	0.1	5.2	**0.04** ^*^

*Note:* FPL, federal poverty line; NHANES, National Health and Nutrition Examination Survey; CI, confidence interval; LL, lower limit; UL, upper limit; SNAP, Supplemental Nutrition Assistance Program.

a Analyses were conducted using survey procedures that take into account the complex survey design.

b Model 1 is adjusted for total energy intake, age, and sex.

c Model 2 is adjusted for total energy intake, adolescent age, sex, race/ethnicity, vigorous recreational activity, moderate recreational activity, sedentary time, household respondent education, marital status, income (linear and quadratic term), and household food security status.

* Statistically significant estimates at alpha = 0.05 are bolded.

## Discussion

In this national sample of lower-income U.S. adolescents aged 12–19 years, the adjusted mean UPF intakes for all lower-income adolescent groups were high, ranging from 63.2% to 65.9% of TEI, which is higher than UPF intake in lower-income U.S. adults.^(12)^ The association between food insecurity and UPF intake was not statistically significant; however, we found that SNAP participation was associated with somewhat higher UPF intake. To our knowledge, this is the first study to rigorously and quantitatively analyse the associations of household food security status and SNAP participation with UPF intake among lower-income U.S. adolescents, and this unique research can have important public health implications. Although this association is cross-sectional, it suggests the need for further research to assess SNAP policies and optimise their impact on public health nutrition.

The high levels of UPF intake across our sample may explain why we did not find a statistically significant association between food insecurity and UPF intake in lower-income U.S. adolescents. The lack of association between food insecurity and UPF intake in the current analysis differs from previous studies. Among lower-income adults in NHANES, food insecurity was associated with higher UPF intake.^(12)^ This finding may have been more easily detected because lower-income adults with food security had UPF intakes lower^(12)^ than the national average.^(8)^ In contrast, it is possible our study did not detect an association due to high UPF intake in all lower-income adolescents, regardless of food security status (unadjusted mean = 66.1%, standard error = 0.5%). In a study focused on food advertisements and UPF intake with food insecurity as a potential effect modifier (i.e. not the main exposure variable), Chiong et al. noted higher UPF intake in U.S. adolescents at risk for food insecurity (measured in their study with a two-item screener with 1+ affirmative responses indicating ‘at risk for experiencing food insecurity’).^(33)^ However, because this information was not the main focus of their study, there was no direct examination of an association between food insecurity and UPF intake. Our finding is different from some researchers who have found an association between food insecurity and poor dietary intake in U.S. children and/or adolescents.^(34,35)^ However, Duke utilised a sample from Minnesota^(34)^ rather than a national sample, so the findings may not be directly comparable. Jun et al. examined ages 1–11 years in addition to adolescents and found significant differences by food security status only with micronutrients,^(35)^ which our study does not examine. Meanwhile, our study aligns with Rossen et al., who found little evidence of an association when looking at food groups and specific dietary components.^(36)^ While further research should be conducted, it is possible that U.S. adolescents’ overall dietary intake is poor across food security levels; the poor intake may stem from eating behaviours more common in this age group, which may promote less healthful intake independent of food security status.

For adolescents, the health repercussions of high UPF intake are significant and may grow over time if high intake continues into adulthood. Compared to less processed foods, UPFs produce lower satiety^(37)^ and have been associated with binge-eating.^(38)^ They have also been shown to be potentially addictive neurologically and behaviourally.^(39)^ This is a particularly negative exposure for the adolescent brain^(40)^ that is still developing and for individuals experiencing stress.^(41)^ Accordingly, for adolescents experiencing food insecurity, which itself is a stressor,^(42)^ the negative consequences of high UPF intake could be especially harmful. In other words, while UPF intake may be similar for all lower-income adolescents, the addictive potential could be worse for adolescents experiencing food insecurity and may lead to high UPF consumption in the future. Finally, as dietary preferences and habits form through childhood and adolescence, excessive consumption of UPFs could create poor future dietary habits. Therefore, the finding of high UPF intake in all lower-income adolescents has public health ramifications as it could increase the risk of nutrition-related diseases in later adulthood.

Meanwhile, the finding for the association between household SNAP participation and higher UPF intake in U.S. adolescents is consistent with prior studies.^(22–24,43)^ First, a study of lower-income U.S. adults found a 1.7% higher UPF intake with SNAP participation.^(12)^ Although the following studies in children and/or adolescents did not examine UPF intake, their overall findings were aligned with our results. For example, among children and/or adolescents, SNAP participation has been associated with higher SSB intake,^(43)^ worse diet quality as measured via the Alternate Healthy Eating Index^(22)^ and HEI,^(23)^ and higher intake of processed meat.^(24)^ Overall, our study builds upon previous evidence by showing specifically that UPF intake — not just dietary components or food groups in general — is high in lower-income adolescents from SNAP-participating households. Although the associations between food insecurity, SNAP participation, and UPF intake in adolescents need further exploration, all lower-income adolescents (as well as the general U.S. population) could benefit from reduced UPF intake. Finally, it is possible that SNAP could serve as an important public health intervention to improve dietary intake in low-income families.

It is notable that adolescents who come from households that participate in SNAP are more likely to consume UPFs. Research has shown that even with SNAP benefits, most participants struggle to follow a healthy diet due to cost and lack of cooking time.^(44)^ While SNAP participation has been shown to improve food insecurity,^(45)^ it might unintentionally lead to more UPF purchases due to cost and convenience.^(44)^ Thus, it is possible that some households might improve their food security status through SNAP participation, but this change may also increase their UPF intake due to the barriers of money and time. While this analysis was cross-sectional and cannot determine if the initiation of SNAP participation improved food security and increased UPF intake, longitudinal studies could examine this question.

Likewise, future research should corroborate these associations as well as examine potential public health interventions that can improve UPF intake in all lower-income U.S. adolescents and adolescents from SNAP-participating households. For example, similar research that examines the association between adolescent self-reported food insecurity and UPF intake might be helpful as self-reported food insecurity (rather than household) may provide a more accurate assessment of food insecurity experienced by the adolescent and may be more closely linked to adolescent UPF intake. Longitudinal analyses that examine the association of food insecurity and SNAP participation with UPF intake from adolescence to adulthood could inform how UPF intake changes with age and when to intervene to improve UPF intake in certain sub-populations. Similarly, qualitative work that examines the behavioural, neighbourhood, and social influences on the association between household SNAP participation and higher adolescent UPF intake could be helpful to understand contextual factors and inform the development of interventions.

Several existing policies and programmes could serve to improve nutritional quality for adolescents from households participating in SNAP. Research has shown that a fruit/vegetable incentive programme for lower-income families with children improved the purchasing of those foods.^(46)^ The Healthy Incentives Pilot^(47)^ and Double-up Food Bucks^(48)^ have been shown to improve dietary intake in SNAP participants. Access to a new neighbourhood supermarket has also been shown to improve SNAP participants’ dietary quality.^(49)^ Research has shown that when children that participate in SNAP acquire food for free in a school setting, the nutritional quality of those foods tends to be higher,^(50)^ so it is possible that the promotion of unprocessed or minimally processed school-based free food acquisitions could be beneficial for UPF intake in adolescents from SNAP-participating households. Finally, even with SNAP benefits, cost^(44)^ has been cited as a barrier to following a healthy diet for SNAP participants, so perhaps higher SNAP benefits could ameliorate this association. However, while some of this research has been conducted in children or includes households with children, more research should investigate how adolescents specifically may respond to these interventions. If proven effective, numerous options could improve UPF intake in lower-income adolescents from SNAP-participating households and the broader U.S. population.

This paper has several strengths. It is unique in its examination of food insecurity, SNAP, and UPF intake in lower-income U.S. adolescents, an understudied research area of high public health importance. Second, our analysis utilises a large national sample. Due to the usage of national data, the findings are broadly applicable. We acknowledge the limitations of this study. First, this analysis used cross-sectional data, so causal inference cannot be drawn. Second, 24-h dietary recalls are prone to recall bias,^(51)^ and two recalls (compared to more than two) may be less accurate for estimating long-term dietary intake. Measurement of UPF intake could be moderately impacted by social desirability bias, and there could be some misclassification of UPF intake due to limited data from NHANES on food processing. Third, the exposures of household food insecurity and household SNAP participation might be experienced differently by adolescents compared to other household members. Finally, as with any observational study, it is possible that there is unmeasured/residual confounding. Nonetheless, this paper contributes important new information to the literature on food insecurity, SNAP participation, and adolescent dietary intake.

In summary, household SNAP participation — but not household food insecurity — was associated with slightly higher UPF intake in a national sample of lower-income U.S. adolescents aged 12–19 years. Despite this association, UPF intake was high for all lower-income adolescents. Policies and programmes that reduce UPF intake for all lower-income U.S. adolescents would be highly beneficial. While SNAP has been shown to improve food security status,^(52)^ it is important to consider how it might impact dietary intake. This paper has highlighted an important and timely public health issue that adolescents from households participating in the programme report high consumption of foods that are potentially addictive^(39)^ and known to increase chronic disease risk.^(4,5)^ Further research into this topic and greater investment in effective public health interventions and policies to promote healthful dietary quality among lower-income adolescents are warranted.
